# Substantial involvement of TRPM7 inhibition in the therapeutic effect of *Ophiocordyceps sinensis* on pulmonary hypertension

**DOI:** 10.1016/j.trsl.2021.03.004

**Published:** 2021-03-07

**Authors:** KEIZO HIRAISHI, LIN HAI KURAHARA, JIANLIN FENG, AYA YAMAMURA, YUANYUAN CUI, EIJI YAHIRO, HIROYASU YOKOMISE, TETSUHIKO GO, KAORI ISHIKAWA, NAOYA YOKOTA, ATSUSHI FUJIWARA, MIKI ONITSUKA, KOHTARO ABE, SHOJI OHGA, TORU SATOH, YASUMASA OKADA, LIXIA YUE, RYUJI INOUE, KATSUYA HIRANO

**Affiliations:** Department of Cardiovascular Physiology, Faculty of Medicine, Kagawa University, Miki-cho, Kita-gun, Kagawa, Japan; Department of Physiology, Fukuoka University School of Medicine, Johnan-ku, Fukuoka, Japan; Department of Cell Biology, University of Connecticut Health Center, Farmington, Connecticut; Department of Physiology, Aichi Medical University, Nagakute, Aichi, Japan; School of Basic Medical Sciences & Shanxi key Laboratory of Brain Disorders, Xi’an Medical University, Xi’an, China; Fukuoka University Medical Education Center, Fukuoka University School of Medicine, Fukuoka University, Johnan-ku, Fukuoka, Japan; Department of General Thoracic Surgery, Faculty of Medicine, Kagawa University, Kita-gun, Miki-cho, Kagawa, Japan; Department of General Medicine, Faculty of Medicine, Kagawa University, Kita-gun, Miki-cho, Kagawa, Japan; Department of Pathology, School of Medicine, Fukuoka University, Fukuoka, Japan; Department of Cardiovascular Medicine, Kyushu University Graduate School of Medical Sciences, Higashi-ku, Fukuoka, Japan; Faculty of Agriculture, Kyusyu University Professor Emeritus, Kasuya-gun, Fukuoka, Japan; Division of Cardiology, Department of Medicine, Kyorin University School of Medicine, Tokyo, Japan; Division of Internal Medicine and Laboratory of Electrophysiology, Murayama Medical Center, Tokyo, Japan.

## Abstract

*Ophiocordyceps sinensis* (OCS), an entomopathogenic fungus, is known to exert antiproliferative and antitissue remodeling effects. Vascular remodeling and vasoconstriction play critical roles in the development of pulmonary hypertension (PH). The therapeutic potential of OCS for PH was investigated using rodent PH models, and cultured pulmonary artery endothelial and smooth muscle cells (PAECs and PASMCs), with a focus on the involvement of TRPM7. OCS ameliorated the development of PH, right ventricular hypertrophy and dysfunction in the monocrotaline-induced PH rats. The genetic knockout of TRPM7 attenuated the development of PH in mice with monocrotaline pyrrole-induced PH. TRPM7 was associated with medial hypertrophy and the plexiform lesions in rats and humans with PH. OCS suppressed proliferation of PASMCs derived from the PH patients. Ethanol extracts of OCS inhibited TRPM7-like current, TGF-β2-induced endothelial-mesenchymal transition, IL-6-induced STAT3 phosphorylation, and PDGF-induced Akt phosphorylation in PAECs or PASMCs. These inhibitory effects were recapitulated by either siRNA-mediated TRPM7 knockdown or treatment with TRPM7 antagonist FTY-720. OCS and FTY-720 induced vasorelaxation in the isolated normal human pulmonary artery. As a result, the present study proposes the therapeutic potential of OCS for the treatment of PH. The inhibition of TRPM7 is suggested to underlie the therapeutic effect of OCS.

## INTRODUCTION

Pulmonary arterial hypertension (PAH) is characterized by a progressive increase in pulmonary vascular resistance due to vascular remodeling, vasoconstriction and thrombus formation in the distal pulmonary arteries (PAs).^1–3^ Currently, 5 classes of drugs, prostacyclin analogues, prostacyclin receptor agonists, endothelin receptor antagonists, phosphodiesterase-5 inhibitors and soluble guanylate cyclase stimulators, are clinically used for the treatment of PAH. Calcium channel blockers are also used but in a limited situation when the patients show a significant immediate hemodynamic response to pulmonary vasodilators.^4^ Despite significant advancement of the therapeutic strategies, the pathogenesis of PAH still remains poor.^5^ Therefore, there is an urgent need to develop new therapeutic methods, especially those targeting the process of vascular remodeling, in order to further improve the prognosis of PAH patients.

*Ophiocordyceps sinensis* (OCS), an entomopathogenic fungus, is used for the treatment of chronic hepatitis, hypertension, erectile dysfunction, inflammation and malignant tumor, in a traditional Chinese medicine.^6,7^ OCS and its relative fungi have been shown to attenuate inflammation and fibrosis in some animal models, such as CCl_4_-induced liver fibrosis, bleomycin-induced pulmonary fibrosis and renal fibrosis induced by ureteral obstruction, partly by inhibiting epithelial-mesenchymal transition.^8–10^ OCS also inhibits proliferation in leukemic cells, artificial culture extracts of *Paecilomyces hepiali* (same species as OCS) are encapsulated for oral administration, and clinical studies are conducted.^11,12^ Vascular remodeling in PAH involves hypertrophy and fibrosis in 3 layers of pulmonary arteries.^13^ The endothelial-mesenchymal transition (EndoMT) provides an important source of *α*-smooth muscle actin (*α*-SMA)-expressing mesenchymal-like cells, which contribute to intimal and medial hypertrophy and fibrosis. The proliferation and reduced apoptosis of smooth muscle cells lead to medial hypertrophy. It is therefore anticipated that OCS will attenuate vascular remodeling in PAH.

FTY-720 is synthesized by the chemical modification of myriocin, a product of *Isaria sinclairii*, another species of entomopathogenic fungus, which is closely related to OCS.^14^ FTY-720 exerts an immunosuppressive effect and is therefore currently use for the treatment of multiple sclerosis.^14^ There are multiple targets of the pharmacological effects of FTY-720; among them, is a transient receptor potential channel, subfamily M, member 7 (TRPM7).^15–17^ Ample evidence demonstrates that several TRP channels participate in the pathophysiological processes of the cardiovascular system, including pulmonary circulation.^18–20^ TRPM7 has been shown to contribute to inflammation, fibrosis, cell migration and cell proliferation, and thereby plays an important role in vascular and cardiac remodeling and cardiac fibrosis.^21,22^ The knockdown of TRPM7 attenuates the shear stress-mediated increases in [Ca^2+^]_i_ in normal and IPAH-derived PASMCs.^23^ TRPM7 is involved in PDGF-BB-induced proliferation via the modulation of Akt kinase.^24,25^ Moreover, TRPM7 is known to modulate STAT3 phosphorylation in glioma stem cells,^26^ while STAT3 phosphorylation has been implicated in proliferative and survival phenotypes of cells comprising plexogenic lesions downstream of IL-6 or PDGF signaling.^27^ TRPM7 also contributes to the epithelial-mesenchymal transition in cancer.^28–33^ Thus, the therapeutic effect of OCS on PAH, if any, could be attributed, at least in part, to the inhibition of TRPM7. However, the inhibitory effect of OCS on TRPM7 remains to be elucidated.

The present study aimed to elucidate the therapeutic potential of OCS for the treatment of PAH. OCS is hypothesized to revert EndoMT, inhibit proliferation of pulmonary artery smooth muscle and mitigate the increased contractility of pulmonary artery, at least in part, by inhibiting TRPM7 activity. The present study addressed these hypotheses using a monocrotaline (MCT) -induced PAH rat model as well as the mice invalidated for the trpm7 gene. Cultured HPAECs and HPASMCs derived from patients with idiopathic PAH (IPAH) were investigated to explore the underlying cellular mechanisms in vitro.

## MATERIAL AND METHODS

### Fungal strain.

*Ophiocordyceps sinensis* (OCS), the fungal strain (Accession number KUMB108) used in this study, was acquired from the mushroom culture bank at the Laboratory of Forest Resources Management, Kyushu University. The genetic analysis showed that the internal transcribed spacer (ITS) sequence of this strain is identical to that seen in *Paecilomyces hepiali* strain Ph-4Qinghai (NCBI Gene bank EF555097.3).^34^ Prior to 2009, *Paecilomyces hepiali* strain Ph-4Qinghai was registered as *Ophiocordyceps sinensis* (OCS) Ph-4Qinghai.

### Preparation of OCS for experimental use.

A solid substrate (40 g brown rice, 0.325 g glucose, 0.65 g sucrose, 2 g peptone and 65 mL corn steep liquor) was used to grow OCS mycelia. The fully colonized substrate was freeze-dried and ground to powder using a Waring blender. In the animal experiments, 1 g of freeze-dried powder was suspended in 20 mL hot water (95°C) and cooled to room temperature and then administered daily by gavage at 0.75 g OCS/kg body weight/day. In the experiments with cultured cells, 1 g of freeze-dried powder was suspended in 20 mL ethanol, sonicated for 5 minutes and vortexed for 1 hour at room temperature. After centrifugation at 3000 rpm for 5 minutes, supernatant was collected as the first extract. The precipitate was further extracted with 10 mL of ethanol, and the supernatant obtained after centrifugation was collected as the second extract. The first and second extracts were combined to make the original OCS ethanol extract. The concentrations of OCS were expressed as dilution rate by volume of the original extract in the working solutions.

### Chemicals.

Monocrotaline (MCT) was purchased from Sigma-Aldrich Chemical Co., (St. Louis, MO). MCT was dissolved in 1 N HCl and at last adjusted pH to 7.4 by 1 N NaOH on each use. MCT pyrrole (MCTp) was purchased from Santa Cruz (Santa Cruz, CA) and dissolved in dimethylformamide. The stock solution of MCTp was kept at −80°C until use. Recombinant human TGF-*β*2 was purchased from Fujifilm Wako Pure Chemical (Osaka, Japan). Stealth RNAi of TRPM7 (M7 si1: HSS123307, M7 si2: HSS182662, M7 si3: HSS182663) and negative control siRNA were obtained from Invitrogen (Carlsbad, CA). FTY-720 was purchased from Cayman Chemical (Ann Arbor, MI) and dissolved in dimethyl sulfoxide. Antibodies against phospho-Smad2 (#3108, Cell Signaling Technology, Danvers, MA), anti-N-cadheline (#ab98952 Abcam, Cambridge, UK), anti-VE-cadheline (#ab33168 Abcam, Cambridge, UK), STAT3 (#9139, Cell Signaling Technology), phospho-STAT3 (#9138, Cell Signaling Technology), *α*-SMA (#ab32575, Abcam, Cambridge, UK), CD31 (#77699, Cell Signaling Technology), vWF (#ab6994, Abcam), TRPM7 (#ab85016, Abcam), Phospho-Akt (Ser473) (#4060, Cell Signaling Technology), Connexin 43 (#ab11370, Abcam) and *β*-actin (#ab6276, Abcam) were used for the immunoblotting and immunostaining experiments.

### Approval of animal experiments.

The study protocols of all animal experiments were approved by the institutional Animal Use and Care committees at Fukuoka University and University of Connecticut School of Medicine, and conducted in accordance with the institutional and NHI guidelines for the care and use of laboratory animals.

### Monocrotaline (MCT)-induced PH model rats.

Sprague-Dawley male rats weighing 240–280 g received a single subcutaneous injection of 60 mg MCT/kg body weight on day 0. Thereafter, they were housed under ambient conditions with ad libitum access to food and water. OCS (0.75 g/kg body weight/day) was orally administered from day 11. The evaluations of PH were performed on day 21. After echocardiographic evaluations, the animals were euthanized by cervical dislocation. The heart and lungs were extracted for further evaluations. The Fulton index, the ratio of the weight of the right ventricular wall to the weight of the left ventricular free wall with the interventricular septum (RV/(LV + S)) was obtained from the excised hearts.

### Tissue samples of Sugen 5416- and hypoxia (SuHx)-induced PH model rats.

Paraffinized lung and right ventricle sections and tissue lysate were prepared from the SuHx-induced PH model rats, as previously described.^35^

### TRPM7-knockout mice and the MCT-pyrrole (MCTp)-induced PH model.

All experiments using TRPM7 knockout mice were performed at the University of Connecticut School of Medicine Health Center, Farmington, CT (animal protocol#: TE-101918–0821). TRPM7^fl/fl^ mice were kindly provided by Dr. D. Clapham, Howard Hughes Medical Institute, Boston, MA.^36^ Six-week-old male Trpm7^fl/fl^ mice were crossed with Rosa26-Cre^ERT2^ mice, then gene deletion was induced by the oral administration of tamoxifen (10 mg/kg body weight, 3 times with a 2-day interval).^37^ Successful gene knockout was confirmed by genotyping and Western blotting as previously described.^36^ MCTp (5 mg/kg body weight) was intravenously administered via the tail vein on day 0. The daily oral administration of OCS (0.75g/kg body weight/day) started 11 days after the injection of MCTp. The evaluations of the pathophysiology of PH were performed on day 14. After hemodynamic evaluations, the animals were euthanized, and the heart and lungs were extracted for further evaluations.

### Hemodynamic evaluation of MCTp-induced PH mice.

The mice were anesthetized by inhalation of isoflurane (1.5% for induction and 1.0% for maintenance via ventilator) at a flow rate of 2–3 L/min. The right ventricular pressure of the mice was measured using a pressure catheter (1.2 F, Transonic System Inc., Ithaca, NY). The pressure catheter was inserted directly into the right ventricle, and connected to the ADVantage PV acquisition system (Transonic System Inc.).

### Echocardiography in rats.

Rats were anesthetized in an anesthesia induction chamber with vaporized isoflurane (1.5% for induction and 1.0% for maintenance) at a flow rate of 2–3 L/min. The rats were then put on a temperature-controlled stage and their rectal temperature was monitored under anesthesia. The chest hair was chemically removed, a prewarmed ultrasound gel was spread over the chest wall, and the transthoracic echocardiography was performed with a Logiq E9 system (GE healthcare, Pittsburgh, PA). The heart rate (HR), right ventricular wall thickness (RVWT), right ventricular internal diameter (RVID) and pulmonary artery acceleration time (PAAT) were evaluated. The RVID was measured as the maximal distance from the RV free wall to the septum in an apical 4-chamber view. The PAAT, the time from the onset of blood flow to maximum velocity, was measured from the pulsed-wave Doppler flow velocity profile of the RV outflow tract in the parasternal short-axis view.

### Cell culture.

HPAECs were purchased from Lonza Japan (Chiba, Japan) and grown in EBM-2 medium containing 5% fetal bovine serum and supplements consisting of human epidermal growth factor, hydrocortisone, GA-1000, VEGF, human fibroblast growth factor-B, R3-insulin like growth factor 1, ascorbic acid and heparin. HPASMCs were purchased from Lifeline Cell Technology Ltd. (Oceanside, CA). HPASMCs were cultured in HuMedia-SG containing 5% fetal bovine serum and supplements consisting of human epidermal growth factor, insulin, human fibroblast growth factor-B, glucose, arginine, gentamicin and amphotericin-B. HPAECs and HPASMCs were cultured for 1–2 weeks at 37°C under 5% CO_2_. HPASMCs were subjected to no more than 4 passages, in accordance with the supplier’s instructions. Cells were treated with TGF-*β*2, IL-6 and PDGF-BB in a media containing 0.5% fetal bovine serum. siRNAs were transfected using Lipofectamine 3000 (Life Technologies) according to the manufacturer’s instructions. The cells were subjected to investigations at 48 hours after transfection.

### Real-time PCR analysis.

Total RNA was extracted from the cultured cells using an RNA Extraction Kit (RNeasy; QIAGEN, Venlo, the Netherlands), and target messenger RNAs were amplified by reverse transcriptase-polymerase chain reaction (RT-PCR; QIAGEN). Real-time PCR was performed using the 7500 Fast Real-Time PCR System (Applied Biosystems, Foster City, CA). The protocol was as follows: initial denaturation at 95°C for 20 seconds, followed by 50 cycles of denaturation (95°C, 5 seconds) and annealing/extension (60˚C, 30 seconds). TaqMan Gene Expression Assays (Life Technologies) were used. The TaqMan probes were ACTA2 (*α*-SMA; Hs00426835_g1) and TRPM7 (TRPM7; Hs00918956_m1).

### Western blotting.

Total cell lysates were prepared in RIPA buffer (50 mM Tris-HCl, adjusted to pH 7.6 by Tris base, 150 mM NaCl, 1% Nonidet P40, 0.5% sodium deoxycholate, 0.1% SDS). Lungs obtained from SuHx rats were homogenized in the lysis buffer with a protease inhibitor cocktail. Supplemented with protease inhibitor cocktail sets (Sigma-Aldrich), and then diluted with Laemmli sample buffer containing 5% 2-mercaptoethanol and 1% bromophenol blue before electrophoresis. Proteins were resolved on 10% sodium dodecyl sulfate-polyacrylamide gels and transferred to polyvinyldifluoride membranes. Membranes were blocked with a blocking reagent (Blocking One, Nacalai Tesque Japan) and incubated overnight at 4°C with the primary antibodies against TRPM7, N-cadherin, *α*-SMA, VE-cadherin, CD31, STAT3, P-STAT3, p-Akt and *β*-actin, followed by 45-min incubation with appropriate horseradish peroxidase-conjugated secondary antibodies at room temperature. The immune complex was detected with an ECL Western Blotting Detection System (LAS3000, Fujifilm, Japan). The expression levels were normalized by those obtained under control conditions, as indicated in each figure legend.

### Preparation of HEK293-expressing murine TRPM7.

Stable transformant of HEK293 cells containing the TRPM7-expression vector (HEK-TRPM7) was as previously described.^38^ Cells were grown in DMEM/Ham’s F-12 medium supplemented with 10% FBS, 100 U/mL penicillin, and 100 mg/mL streptomycin at 37°C in a humidity-controlled incubator under 5% CO_2_. The expression of TRPM7 was induced by the addition of tetracycline (1 mg/mL) to the growth medium; experiments were performed 24 hours later.

### Electrophysiological study.

HAPSMCs were serum-starved for 24–48 hours prior to the electrophysiological studies. HAPSMCs and HEK-TRPM7 were dispersed by treatment with 1×TripLE (Thermo Fisher Scientific, Tokyo, Japan) and 0.01% trypsin in PBS, respectively, and transferred into the recording chamber coated with 0.01% poly-L-lysin. TRPM7-like currents were recorded with a whole-cell patch clamp configuration from cells bathed in standard extracellular solution composed of 145 mM NaCl, 5 mM KCl, 2 mM CaCl_2_, 10 mM HEPES and 10 mM glucose, adjusted to pH 7.4 with NaOH. TRPM7-like currents were induced by the introduction of the internal solution composed of 120 mM Cs-aspartate, 20 mM CsCl, 5 mM BAPTA, 1.5 mM CaCl_2_, 10 mM HEPES adjusted to pH 7.2 by NaOH in the case with HPASMCs and the internal solution composed of 115 mM Cs-methanesulfonate, 5 mM ATP-Na_2_, 10 mM EGTA-Cs, and 10 mM HEPES, adjusted to pH 7.2 with CsOH in the case of HEK-TRPM7. The holding potential was set to −60 mV. In some measurements, ramp voltage (from −100 to +100 mV/s) was intermittently applied to the cell using an EPC8 amplifier (HEKA Elektronik, Lambrecht/Pflaz, Germany) in conjunction with an AD/DA converter (LIH8+8, InstruTECH, Longmont, CO) under the control of the Patchmaster software program (v.2 90.1, HEKA Elektronik), at a digitizing rate of 3 kHz after 1 kHz low-pass filtering. For consecutive recordings (>1 minute), PowerLab 4/25 (AD Instruments, New South Wales, Australia) was used with 50 Hz low-pass filtering and 100 Hz digitization. Patch electrodes were prepared by an automatic puller (Sutter Instrument, Novato, CA) and the tip of the pipette was heat-polished in a micro-forge to give an input resistance of 4–6 MV with Cs-based internal solution.

### Clinical specimens for histological evaluations.

Frozen lung tissues of the patients with IPAH and the control without pulmonary artery remodeling were obtained at autopsy in Kyorin University and Fukuoka University, respectively (Supplementary Table 1). The control tissues were obtained from the patients with no apparent lung diseases. The clinical study protocol was approved by the Institutional Review Boards of the Faculty of Medicine, Kyorin University, the Faculty of Medicine, Fukuoka University and the Faculty of Medicine, Kagawa University.

### Histological evaluations.

The rat lungs were inflated with PBS containing 1% formalin plus 0.5% agarose via the trachea and then fixed with 10% formalin in neutral buffer solution overnight. Lungs from PAH and non-PAH patients (postmortem), and hearts and lungs from experimental animals were fixed in 10% buffered formalin and embedded in paraffin. The 4-*µ*m-thick sections were obtained for routine hematoxylin-eosin (HE) and Masson trichrome (MT) staining. The fibrosis score of the rat hearts was obtained by an independent pathologist who quantified the intensity of the blue color of MT staining in a blinded manner using a free software program (Image J, NIH, Bethesda, MD). The muscularization index was determined by *α*-SMA staining of mouse lung sections. Nonmuscularized, partially muscularized and totally muscularized arteries were defined by positive staining of *α*-SMA in <25%: nonmuscularized (N), 25% 75%: partially muscularized (P), and >75% of the circumference of the arteriole: fully muscularized (F), respectively. At least 15 arterioles were evaluated for each specimen in a blinded manner.

### Fluorescence staining.

Formalin-fixed, paraffin-embedded rat and mouse lung specimens were deparaffinized and rehydrated, followed by heat antigen retrieval. The cultured cells were fixed in 4% formaldehyde for 15 minutes and permeabilized with 0.3% Triton X-100 in 5% normal goat serum (Wako, Japan). Tissues and cells were incubated with primary antibodies (1:200 dilution) against *α*-SMA and VE-cadherin at 4°C overnight, washed with PBS, and incubated with an Alexa Fluor-conjugated secondary antibody (1:200 dilution; Life Technologies, Carlsbad, California, United States) for an additional 1 hour. DAPI and To-Pro-3 were used to detect nuclei in the tissue specimens and the cultured cells, respectively. Fluorescence images were acquired using a Zeiss LSM 710 confocal microscope with a 40X objective lens (Oberkochen, Germany).

### Preparation of PASMCs derived from IPAH patients.

PASMCs of patients with idiopathic PAH (IPAH-PASMCs) was established as previously reported and used in the present study.^39^ For the control experiment, PASMCs of normal subjects (Normal-PASMCs) were purchased from Lonza (Walkersville, MD). The cells were cultured in Medium 199 supplemented with 10% heat-inactivated fetal bovine serum, 100 U/mL penicillin plus 100 *µ*g/mL streptomycin (Invitrogen/GIBCO, Grand Island), 50 *µ*g/mL D-valine (Sigma-Aldrich) and 20 *µ*g/mL endothelial cell growth supplement (BD Biosciences, Franklin Lakes) at 37°C. The cells were used in the experiments at passages 5 to 10. Three independent lines of IPAH- and Normal-PASMCs were used respectively.

### MTT assay in IPAH-PASMCs.

IPAH- and normal-PASMCs were plated at 1×10^4^ cells per well in 96-well plates and incubated at 37°C for 6 hours before assay. PASMCs were then cultured in the presence and absence of drugs for a further 3 days. The number of viable cells on day 3 was evaluated as an index of cell proliferation, using a Cell Counting Kit-8 (Dojin, Kumamoto, Japan) based on an MTT (3-(4,5-dimethyl-2-thiazolyl)-2,5-diphenyl-2H-tetrazolium bromide) assay. The results were quantified colorimetrically as the absorbance at 450 nm (A_450_) using a Benchmark Plus Microplate Reader and Microplate Manager (ver. 5.2; Bio-Rad Laboratories, Hercules).

### Bromodeoxyuridine (BrdU) incorporation assay in IPAH-PASMCs.

The proliferation of IPAH-PASMCs was evaluated using a Cell Proliferation ELISA, BrdU (colorimetric) kit (Roche Diagnostics, Mannheim, Germany). In brief, cells were plated and cultured for 72 hours, as described in the MTT assay section. The cells were then exposed to 100 *µ*M BrdU for 30 minutes in the presence and absence of 0.1% OCS. The incorporation of BrdU was quantified colorimetrically as absorbance at 370 nm using a SpectraMax M3 (Molecular Devices, San Jose, CA), according to the manufacturer’s instructions. The extent of BrdU incorporation obtained in the presence of 0.1% OCS was assigned a value of 100%.

### Tension measurement of ring preparations of normal human intrapulmonary artery.

The study protocol was approved by the Internal Review Board, the Faculty of Medicine, Kagawa University. The ring preparations of normal human intrapulmonary artery were prepared from the peripheral lung blocks obtained during lobectomy from patients with a clinical diagnosis of lung cancer. The lung specimens were sufficiently distant from and apparently free of cancerous lesions. The intrapulmonary arteries (diameter: 700–1500 *µ*m) were carefully excised and the surrounding lung tissues were carefully removed under a binocular microscope. The excised arterial segments were cut into approximately 3-mm wide ring preparations. The rings were mounted horizontally on a pair of steel wires immersed in 250 *µ*L normal physiological salt solution (PSS) on silicon rubber plates, which were placed on the thermoregulatory unit, which was set to 33°C. One wire was fixed, while the other was connected to the force transducer U gauge (Minebea, Nagoya, Japan). The compositions of PSS were 140 mM NaCl, 5 mM KCl, 1 mM CaCl_2_, 1.5 mM MgCl_2_, 10 mM HEPES and 10 mM glucose (pH 7.4, adjusted with Tris base). High K^+^-PSS was prepared by equimolar substitution of NaCl for KCl. The rings were repeatedly stimulated with 80 mM K^+^-depolarization during an equilibration period. After obtaining steady responses to 80 mM K^+^-depolarization, the experimental protocols were started to examine the relaxant effect of OCS and FTY-720. The endothelium was kept intact. The presence of a functional endothelium was confirmed by the acetylcholine-induced relaxant response. The level of tension was expressed as a percentage by assigning values obtained under resting conditions and those obtained just prior to the application of OCS and FTY-720 of 0% and 100%, respectively.

### Statistical analysis.

Kaplan-Meier curves were generated the SAS 9.4 (Statistical Analysis System) at Fukuoka University. Survival rates were compared across the groups using the Wilcoxon test and the Tukey-Kramer post hoc method using the LIFETEST procedure. The statistical significance of differences between 2 groups was evaluated by Student’s *t* test. Multiple comparison was made by a one-way analysis of variance (ANOVA) followed by Dunnett’s or Tukey-Kramer post hoc test as indicated. *P* values of <0.05 were considered to indicate statistical significance.

## RESULTS

### OCS improves the signs of pulmonary hypertension in MCT-induced PH model rats.

**Fig 1.**
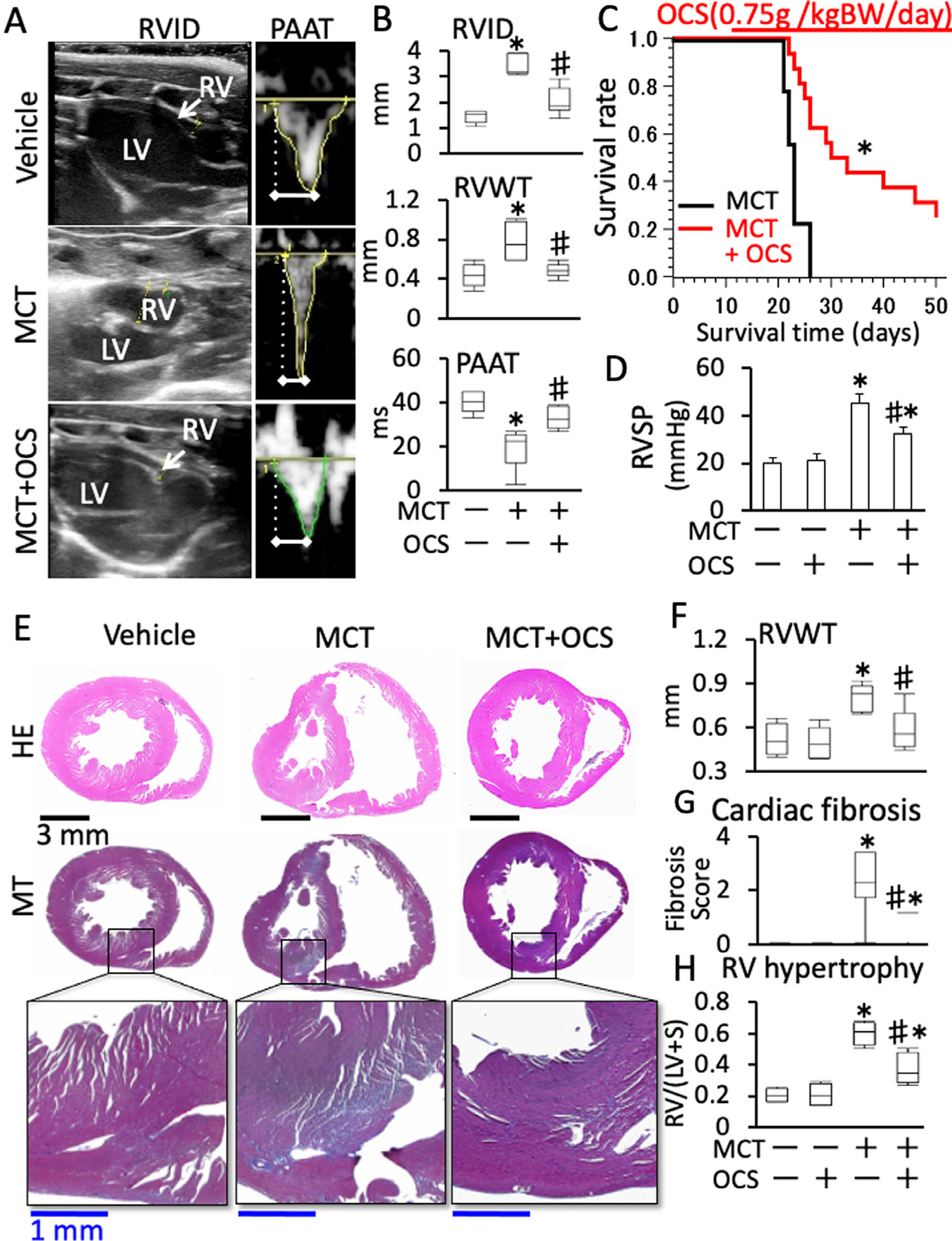
OCS showed ameliorating effects on right ventricular dysfunction and remodeling in MCT-induced PH rats. (A, B) Representative images of echocardiography and Doppler echocardiography (A) and summaries (B; *n* = 5–6) of the hemodynamic indices (right ventricular internal diameter [RVID], right ventricular wall thickness [RVWT] and pulmonary artery acceleration time [PAAT]) in the vehicle, MCT and MCT+OCS rats. *P < 0.05 vs vehicle; #*P* < 0.05 vs MCT, according to ANOVA followed by Tukey-Kramer post hoc test. (C) The survival curves of the MCT-induced PH rats with (MCT-OCS) and without (MCT) the administration of OCS starting from 11 days after MCT injection. *P < 0.05 vs MCT, according to the Wilcoxon test and Tukey-Kramer multiple comparison test (*n* = 9–12) (D) Summary (*n* = 4–6) of the right ventricular systolic pressure (RVSP) of the vehicle, OCS, MCT and MCT+OCS rats. (E) Representative images of hematoxylin-eosin (HE) and Masson’s trichrome (MT) staining of cross-sections of the hearts from the vehicle, MCT and MCT+OCS rats. (F-H) Summaries (*n* = 5–6) of the right ventricular wall thickness (F; RVWT), cardiac fibrosis (G) and RV hypertrophy (H) in the vehicle, OCS, MCT and MCT+OCS rats. * *P* < 0.05 vs vehicle rats (MCT -, OCS -); # *P* < 0.05 vs MCT rats (MCT+, OCS -) according to an ANOVA followed by Tukey-Kramer post hoc test.

We first examined the potential therapeutic benefit of OCS for PH, using MCT-induced PH model rats (Fig 1). The rats were treated with OCS (0.75 g/kg body weight/day) starting from day 11 after MCT injection. Echocardiography performed on day 21 demonstrated that OCS treatment significantly improved the increases in the RVID and RVWT and the decrease in the PAAT, the features of RV dysfunction, in the MCT-induced PH rats (Fig 1A and B). OCS treatment also significantly prolonged the survival of the MCT-induced PH rats and suppressed the increase in the RV systolic pressure (Fig 1C and D). OCS alone had no significant effect on the RV systolic pressure (Fig 1D). Furthermore, OCS treatment significantly attenuated the increase in the RV wall thickness and fibrosis score and the development of RV hypertrophy, as evaluated by the Fulton index, in the MCT-induced PH rats (Fig 1E–H). OCS alone had no significant effect on these values (Fig 1F–H). The expression of TRPM7 in the right ventricle was detected in fibroblast like cells (Supplementary Fig 1). The TRPM7 expression increased in the MCT-induced PH rats, and this increase was attenuated by the treatment with OCS.

### The expression of TRPM7 was increased in the distal PAs in MCT- and SuHx-induced PH model rats.

**Fig 2.**
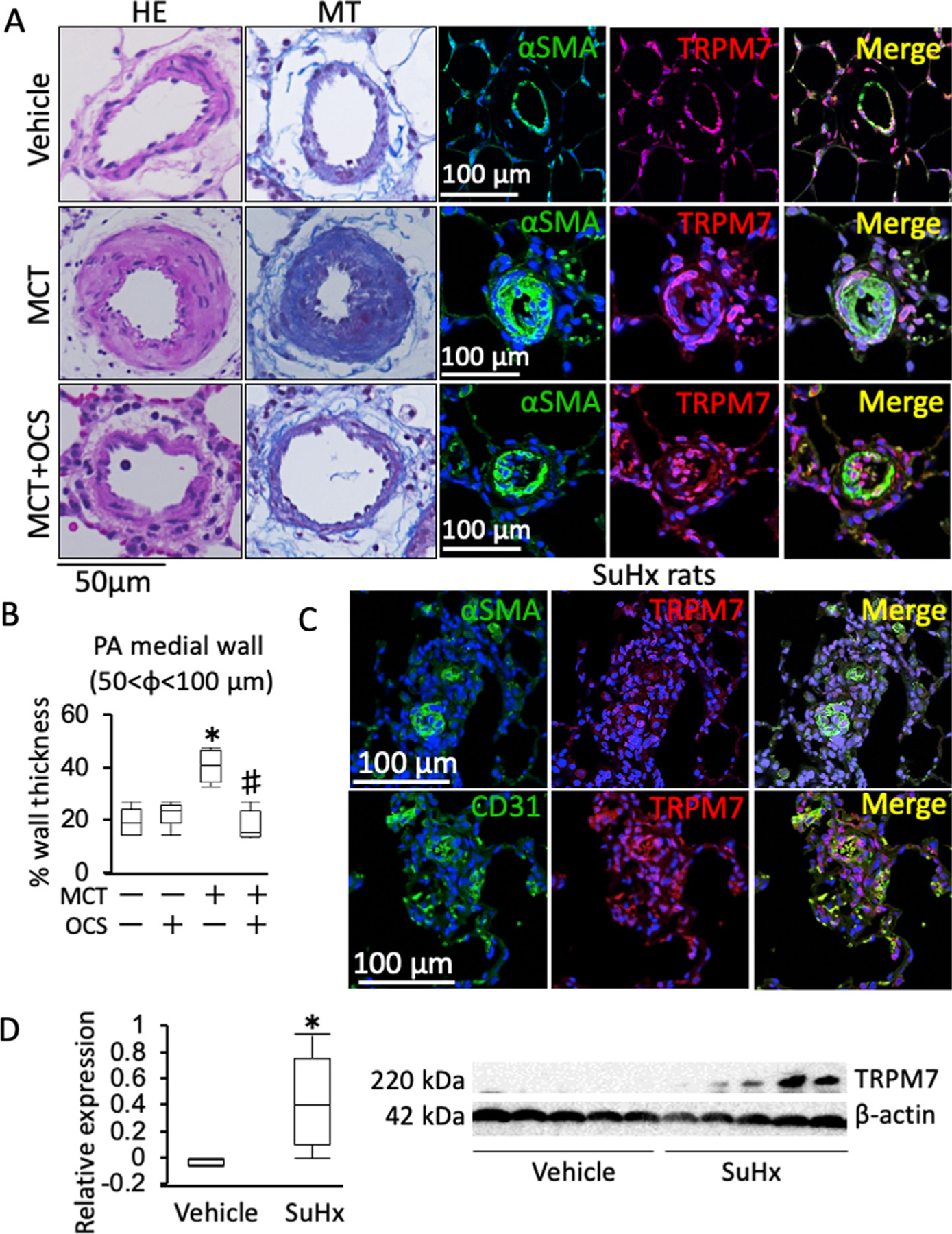
The expression of TRPM7 was increased in the medial layer of the pulmonary artery in MCT- and SuHx-induced PH rats. (A) Representative images of immunofluorescence staining of a-smooth muscle actin (*α*-SMA; green), TRPM7 (red) and DAPI (blue) as well as hematoxylin-eosin (HE) and Masson’s trichrome (MT) staining, as indicated, in the lungs of vehicle, MCT and MCT+OCS (*n* = 5). (B) Summary (*n* = 5) of the medial wall thickness of the pulmonary arteries (diameter, 50–100 *µ*m) of the vehicle, OCS, MCT and MCT+OCS rats. The data are shown in a boxplot (*n* = 5). *P < 0.05 vs vehicle rats (MCT-, OCS -); #*P* < 0.05 vs MCT rats (MCT+, OCS-), according to an ANOVA followed by Tukey-Kramer post hoc test. (C) Representative images of immunofluorescence staining of *α*-SMA (green), TRPM7 (red), CD31 (green) and DAPI (blue) in the lungs of SuHx-induced PH rats (C; *n* = 3). (D) Representative Western blot images and summary of the expression of TRPM7 in the lung of the vehicle and SuHx rats. (*n* = 5)༊*P* < 0.05 vs vehicle rats.

In the control rats, TRPM7 was co-localized with *α*-SMA (Fig 2A). In the MCT-induced PH rats, the medial wall thickness increased with an increase in fibrosis and *α*-SMA immunoreactivity (Fig 2A and B). TRPM7 immunoreactivity increased in association with the increase in *α*-SMA immunoreactivity (Fig 2A). OCS treatment significantly reduced the medial wall thickness, and reduced the immunoreactivity of *α*-SMA and TRPM7 in the MCT-induced PH rats (Fig 2A). In the periluminal plexiform lesions of the SuHx-induced PH rats, TRPM7 immunoreactivity was co-localized with immunoreactivity to both *α*-SMA and CD31 (Fig 2C). In the pulmonary artery of SuHx rats, TRPM7 immunoreactivity was co-localized with those of CD31 or von Willebrand factor (vWF). The extent of this colocalization was greatly increased in SuHx-induced PH than vehicle-treated rats (Supplementary Fig 2A). The level of TRPM7 protein expression in the lung was significantly higher in SuHx rats than vehicle-treated ones (Fig 2D). The protein level of phosphorylated STAT3 in the lungs of SuHx rats was significantly higher than in that of vehicle rats (Supplementary Fig 2B). In the right ventricle of SuHx rats, TRPM7 expression was localized in *α*-SMA positive fibroblasts, and the area of TRPM7 and *α*-SMA being co-expressed was greatly enlarged in SuHx rats compared with the vehicle-treated rats (Supplementary Fig 3).

### TRPM7 deletion attenuates the signs of pulmonary hypertension in MCTp-induced PH mouse.

**Fig 3.**
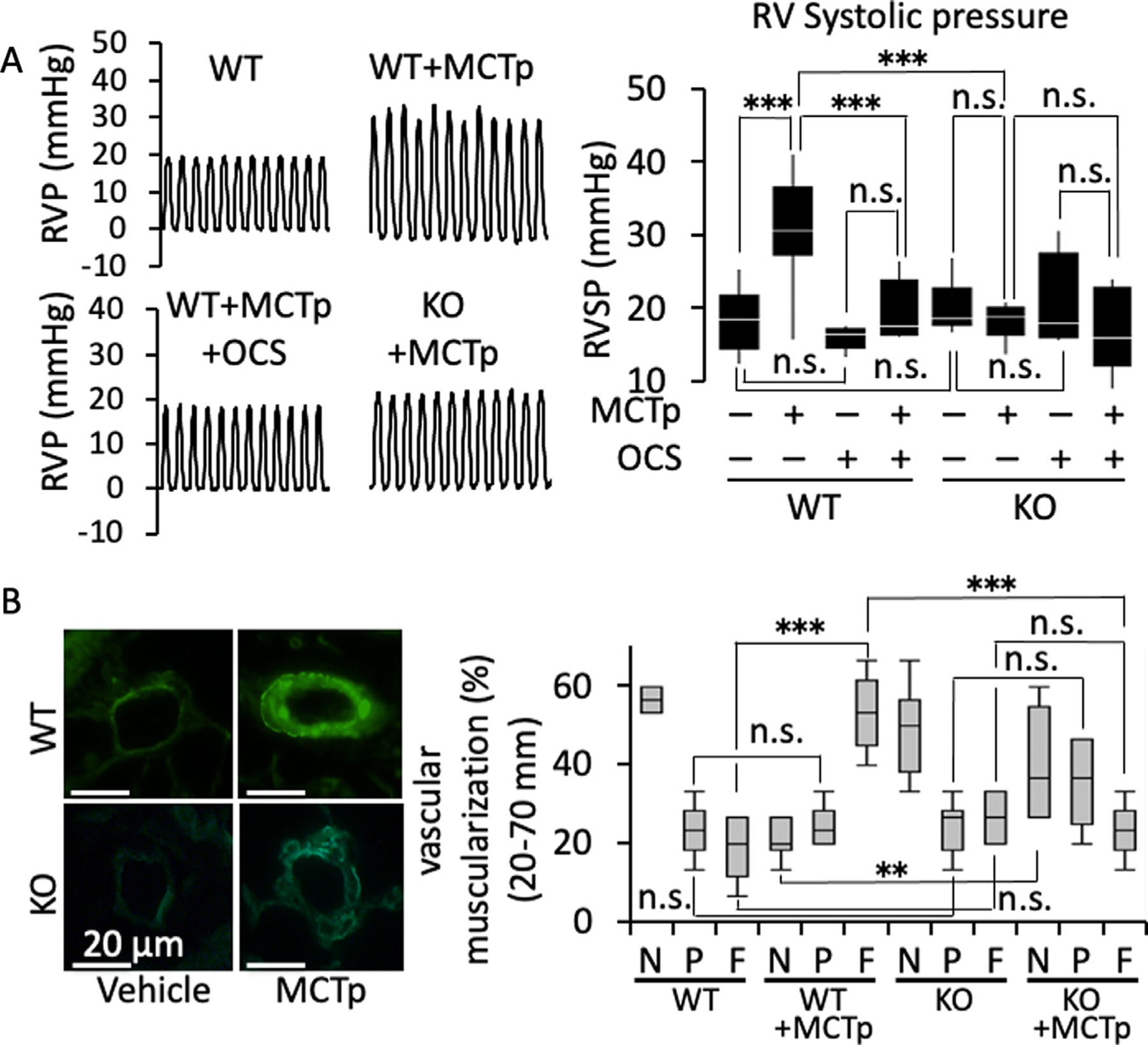
Amelioration of the pathophysiology of MCT pyrrole (MCTp)-induced PH in TRPM7 gene-knockout mice. (A) Representative recordings of the right ventricle pressure (RVP) and a summary (*n* = 5–6) of the systolic RVP (RVSP) values in the wild-type and TRPM7-knockout (KO) mice treated with MCTp and OCS, as indicated. (B) Representative images of immunofluorescence staining of a-smooth muscle actin (green) in the lung specimens of wild-type (WT) and TRPM7-knockout (KO) mice treated with vehicle and MCTp, as indicated. Percentages of nonmuscularized (N), partially muscularized (P) and fully muscularized (F) pulmonary arteries are summarized (*n* = 6). The data are shown in boxplots. *P < 0.05, **P < 0.01 or ***P < 0.001 vs wild-type mice treated with MCTp, according to an ANOVA followed by Tukey-Kramer post hoc test.

To unequivocally substantiate the contribution of TRPM7 to the pathogenesis of PH, TRPM7-knockout mice were utilized (Fig 3). The mouse PH model was generated by treatment with MCTp. The RV pressure and vascular muscularization were evaluated on day 14 after MCTp injection. In wild-type mice, MCTp treatment significantly elevated the RV systolic pressure (Fig 3A). OCS treatment in wild-type MCTp mice and knockout of the TRPM7 gene counteracted the elevation of the RV systolic pressure (Fig 3A). Knockout of the TRPM7 gene had no significant effect on the basal level of RV systolic pressure. OCS had no significant effect on the RV systolic pressure in TRPM7-knockout mice, regardless of whether MCTp treatment was administered (Fig 3A). The PA muscularization induced by MCTp in wild-type mice was significantly inhibited by TRPM7 knockout (Fig 3B).

### OCS inhibits the channel activity of both endogenous and heterologously expressed TRPM7.

**Fig 4.**
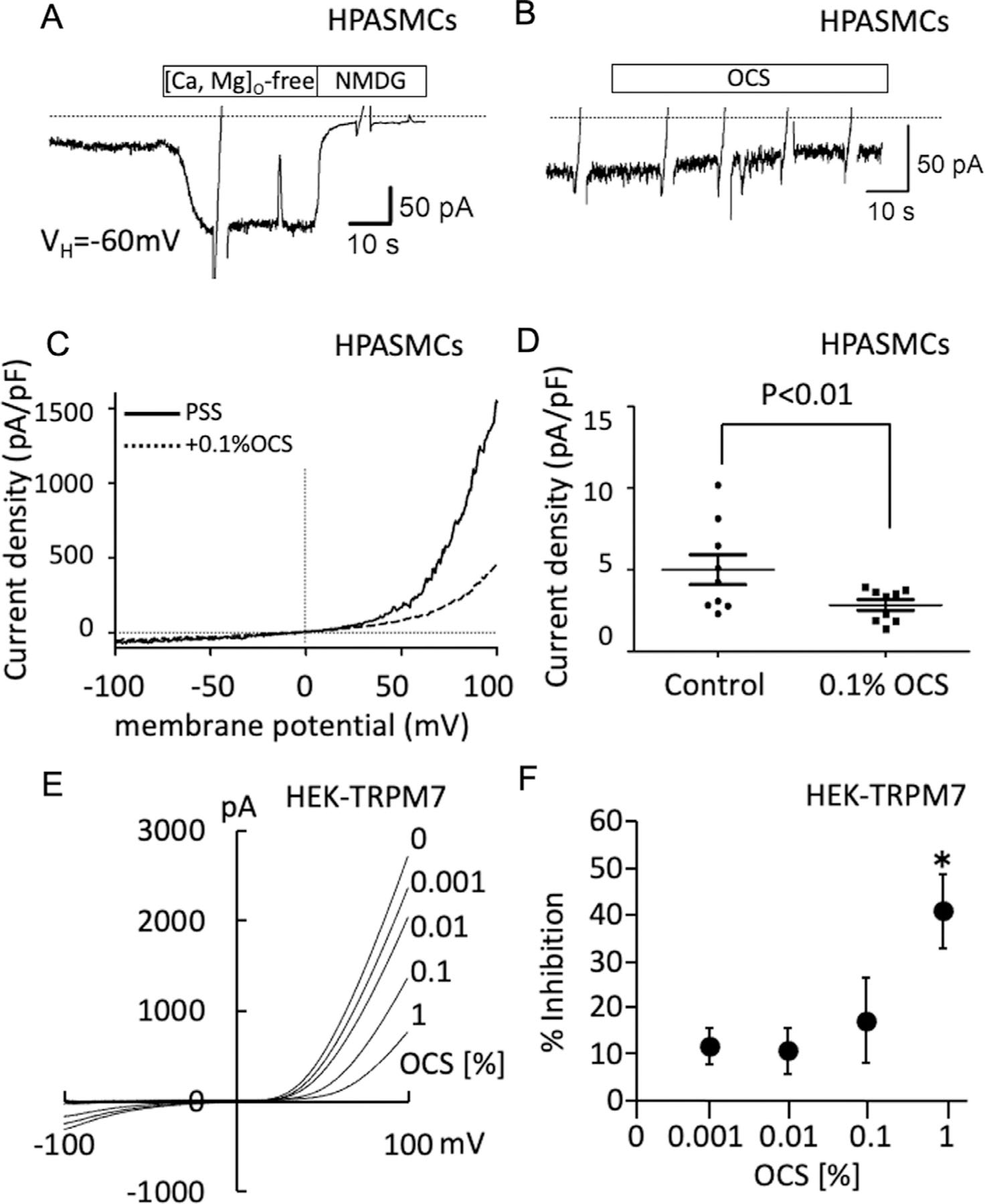
TRPM7-like currents recorded from HPASMCs and HEK-TRPM7. (A) A representative recording of the inward current in HPASMCs bathed in the physiological saline (PSS) and intracellularly perfused with the Mg^2+^, ATP-free Cs-based solution ([Mg, ATP]_i_-free) via a patch pipette. The current was enhanced by the removal of extracellular Ca^2+^ and Mg^2+^ ([Ca, Mg]_o_-free) and inhibited by substitution of external cations with N-methyl, D-glucamine (NMDG). (B) A representative recording showing the inhibition of TRPM7-like current by external application of 0.1% OCS. (C) Representative recordings showing the current-voltage (I-V) relationship of TRPM7-like current before (solid) and after (dashed) 0.1% OCS application. Ramp voltages (−100 to +100mV, 1s) were repeatedly applied to obtain the I-V relationships. (D) A summary of the density of TRPM7-like current observed under the holding potential at −60 mV in the presence and absence of 0.1% OCS. The data are expressed as the mean § S.E.M. with individual data points shown as dots (*n* = 7). The *P* value was obtained by a paired Student’s *t* test. (E) Representative recordings showing the I–V relationship of the currents recorded from HEK-TRPM7, which were bathed in PSS with and without the indicated concentrations of OCS and intracellularly perfused with the Mg^2+^, ATP-free Cs-based solution. (F) Summary of the concentration-dependent inhibitory effects of OCS on the TRPM7-mediated current observed at +100 mV. The data are expressed as the mean § S.E.M. (*n* = 4–6). *, *P* < 0.05 according to an ANOVA followed by Dunnett’s post hoc test.

**Fig 5.**
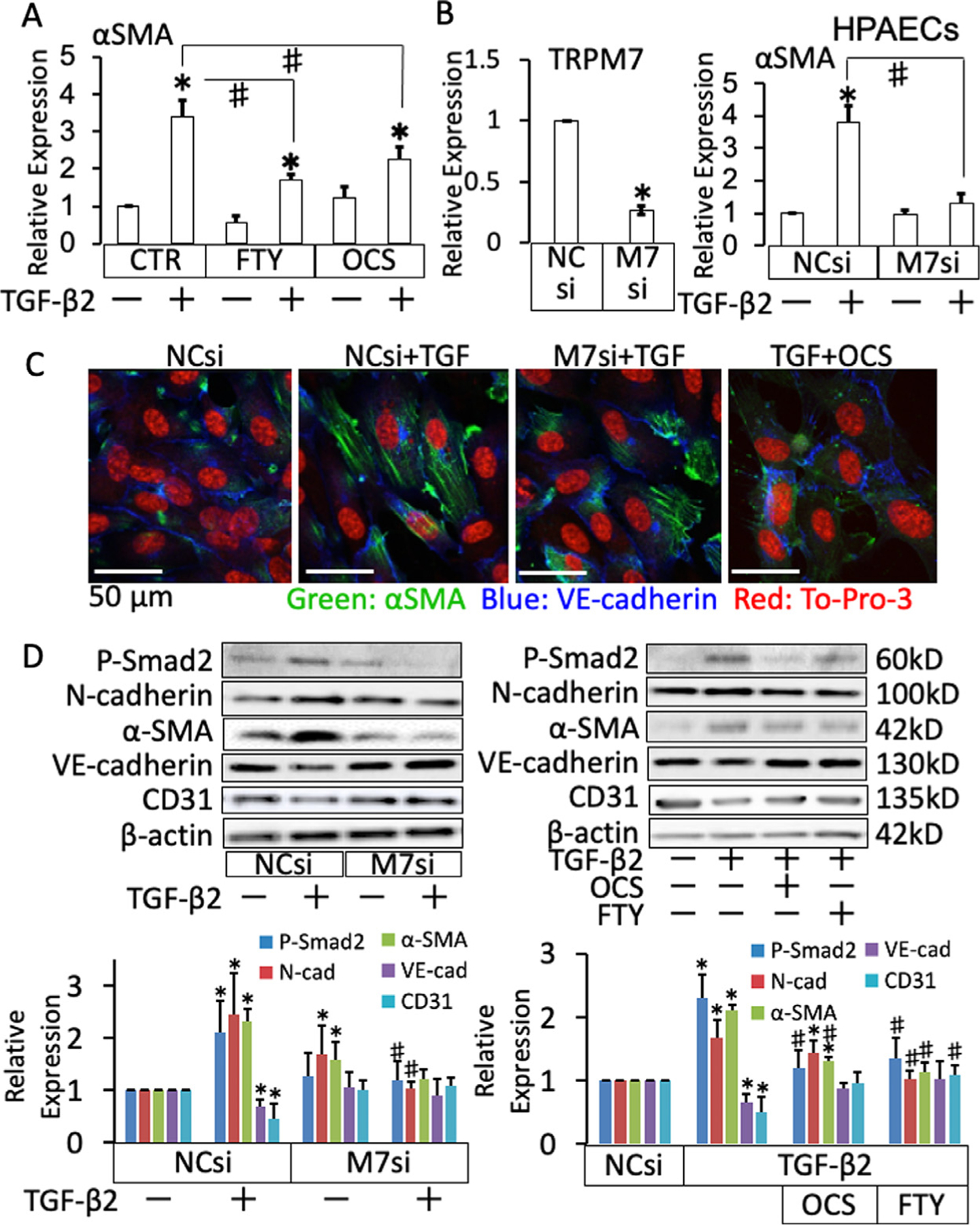
Suppression of the endothelium-mesenchymal transition by the pharmacological and genetic inhibition of TRPM7 in HPAECs. (A, B) Summaries of the real-time PCR analysis to detect the expression of a-smooth muscle actin (*α*-SMA) in HPAECs with and without treatment with 5 ng/mL TGF-*β*2, 1 *µ*M FTY-720 and 0.1% OCS (A) and transfection with negative control (NCSi) and TRPM7-trageted (M7si) siRNA (B), as indicated. B shows a summary of the levels of TRPM7 mRNA. The data are expressed as the mean ± S.E.M (*n* = 4). *P < 0.05 vs control (CTR) or NCsi-transfected HPAECs (NCsi) without TGF-*β*2 treatment; #*P* < 0.05 as indicated, according to an ANOVA followed by Tukey-Kramer post hoc test. (C) Representative images (*n* = 4–7) of fluorescence staining of *α*-smooth muscle actin (*α*-SMA; green), VE-cadherin (blue) and To-Pro-3 (red) in HPAECs transfected with negative control (NCsi) and TRPM7-targeted (M7si) siRNA or treated with 0.1% OCS with and without 5 ng/mL TGF-*β*2, as indicated. (D) Representative chemiluminescence images and summaries of the results of the Western blot analyses (*n* = 4–6) of the expression of mesenchymal markers (N-cadherin and *α*-SMA) and endothelial markers (VE-cadherin and CD31) as well as the phosphorylated form of smad2 and *β*-actin in HPAECs transfected with negative control (NCsi) or TRPM7-trageted (M7si) siRNA or treated with 0.1% OCS and 1 *µ*M FTY-720, with and without treatment with 5 ng/mL TGF-*β*2, as indicated. The data are expressed as the mean ± SD *P < 0.01 vs NCsi or control (CTR), #*P*< 0.01 vs NCsi+TGF-*β*2 for M7si or TGF-*β*2 for OCS or FTY-720, according to an ANOVA followed by Tukey-Kramer post hoc test.

**Fig 6.**
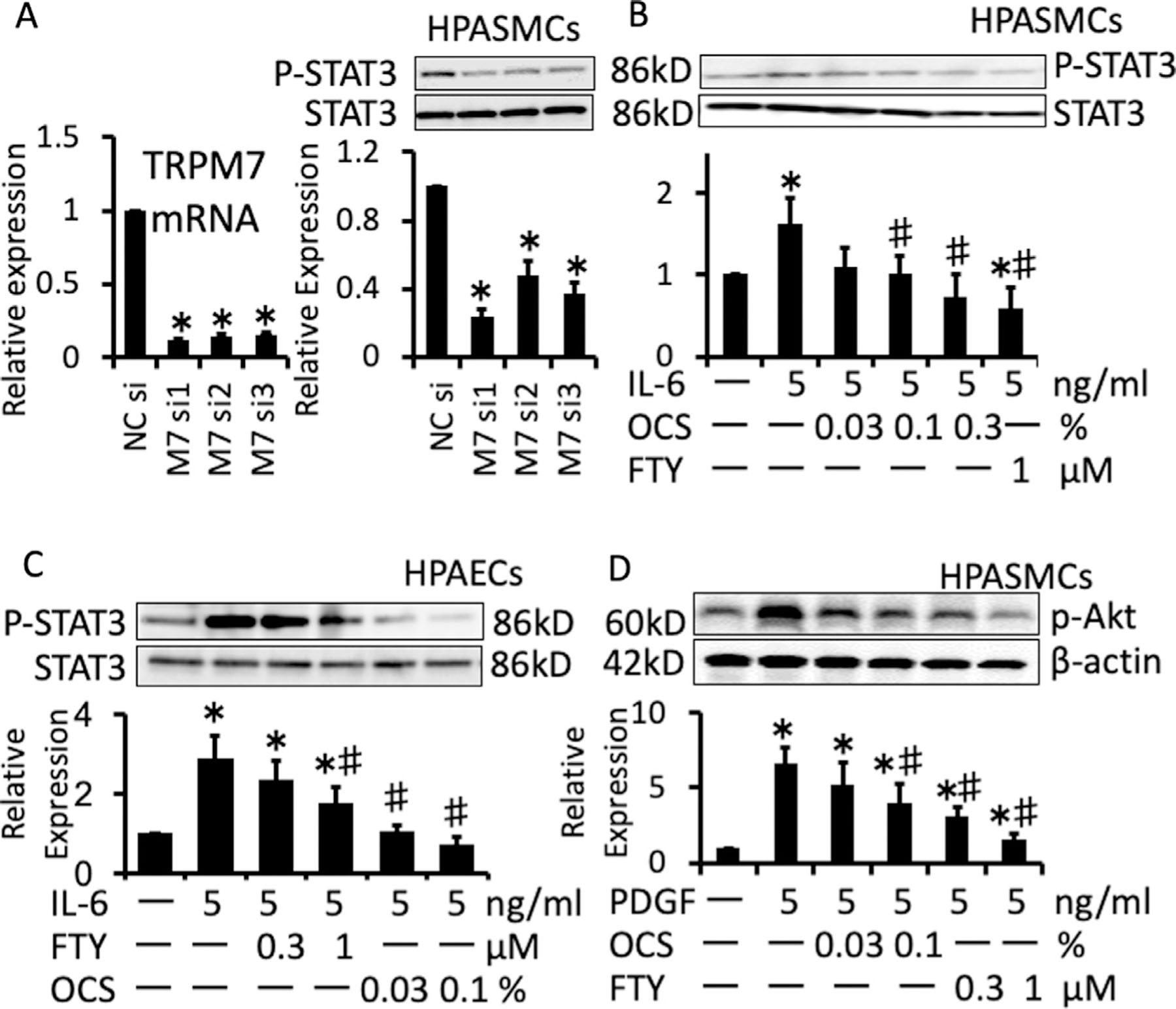
Suppression of STAT3 and Akt phosphorylation by inhibition of TRPM7 in HPASMCs and HPAECs. (A) Summary of the levels of TRPM7 mRNA, and representative chemiluminescence images and summaries of the results of Western blot analyses (*n* = 4) of the basal levels of total (STAT3) and phosphorylated STAT3 (P-STAT3) in HPASMCs transfected with negative control siRNA (NCsi) or 3 different siRNA targeted to TRPM7 (M7si1–3). (B, C) Representative chemiluminescence images and summaries of the Western blot analyses (*n* = 4) of the levels of STAT3 and P-STAT3 in HPASMC and HPAECs treated or untreated with IL-6, OCS or FTY-720 at the indicated concentrations. (D) Representative chemiluminescence images and a summary of the results of the Western blot analysis (*n* = 4) of the levels of Akt phosphorylated at Ser473 (P-Akt) in HPASMCs treated with PDGF-BB, OCS or FTY-720 at the indicated concentrations. The data are expressed as the mean ± SD *P < 0.01 vs NCsi (A) and control (no treatment in B, C, D), #*P* < 0.01 vs IL-6 or PDGF-BB alone, according to an ANOVA followed by Tukey-Kramer post hoc test.

To obtain some mechanistic insight into the therapeutic effect of OCS, a series of experiments with cultured cells were conducted using the ethanol extract of OCS (Fig. 4–6). First, the inhibitory effect of the OCS extract on the TRPM7 channel activity was investigated (Fig 4). Upon internal perfusion of HPASMC with the Mg^2+^-free, ATP-free solution, an inward current rapidly developed, with a current density of approximately 5 pA/pF, under the holding potential of −60 mV (Fig 4A and C). This inward current was greatly enhanced in amplitude by removal of extracellular divalent cations (Ca^2+^, Mg^2+^), almost completely suppressed by total substitution of extracellular cations with N-methyl, D-glucamine (Fig 4A, B), and showed strong outward rectification in response to a voltage ramp spanning between −100 and 100 mV within a 1-s period (Fig 4C). These features are the hallmarks of TRPM7-mediated current.^40^ This TRPM7-like current observed at the holding potential of −60 mV was significantly inhibited by 0.1% OCS and FTY-720 in HPASMCs (Fig 4B–D, Supplementary Fig 4). Consistent with this finding, OCS suppressed the TRPM7-mediated currents in HEK-TRPM7 in a concentration-dependent manner (Fig 4E). Statistically significant inhibition occurred with 1% OCS (Fig 4F).

### Inhibition of TRPM7 activity suppressed the TGF-β2-induced endothelial-mesenchymal transition (EndoMT) in HPAECs.

Since the expression of TRPM7 was detected in CD31-positive cells in SuHx-induced PH rats (Fig 2C), the possible involvement of TRPM7 in EndoMT was investigated using cultured HPAECs (Fig 5). TGF-*β*2 (5 ng/mL) was used to induce EndoMT. Quantitative PCR, immunofluorescence staining and Western blotting showed that TGF-*β*2 increased the expression of mesenchymal markers, *α*-SMA and N-cadherin, and reduced the expression of endothelial markers, VE-cadherin and CD31 (Fig 5A–D). TGF-*β*2 treatment also facilitated the formation of *α*-SMA-decorated stress fibers (Fig 5C). The phosphorylation level of Smad2, a canonical TGF-*β*2 signaling molecule, increased after TGF-*β*2 treatment (Fig 5D). All of these changes were suppressed by treatment with either 0.1% OCS or 1 *µ*M FTY-720, as well as knockdown of the TRPM7 expression by siRNA (Fig 5A–D).

### Phosphorylation of STAT3 and Akt depends on TRPM7 activity.

The involvement of TRPM7 in the phosphorylation of STAT3 and Akt was investigated in HAPECs and HPASMCs, as the activation of STAT3 and Akt signaling has been reported to be involved in the vascular remodeling associated with PAH.^27,41^ The siRNA-mediated knockdown of TRPM7 reduced the basal phosphorylation level of STAT3 in HPASMCs (Fig 6A). Treatment with 0.1% and 0.3% OCS or 1 *µ*M FTY-720 significantly abrogated the phosphorylation of STAT3 induced by 1 ng/mL IL-6 in HPAECs and HPASMCs (Fig 6B and C). Treatment with 0.03% and 0.1% OCS or 0.3 *µ*M and 1 *µ*M FTY-720 suppressed the Akt phosphorylation induced by 5 ng/mL PDGF in HPASMCs (Fig 6D).

### OCS exhibits an antiproliferative effect in IPAH-PASMCs and vasodilation in human PA.

**Fig 7.**
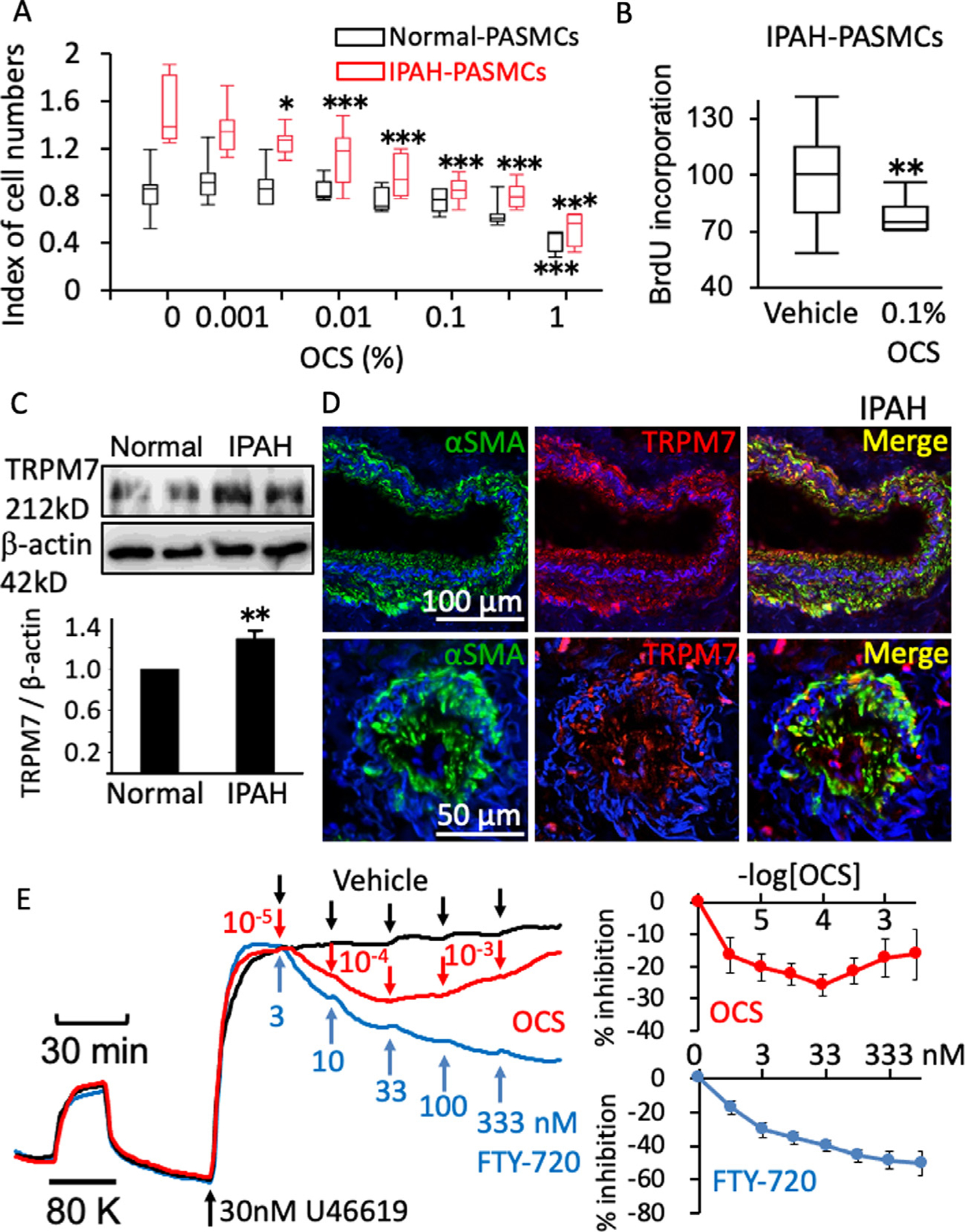
The inhibitory effects of OCS on the enhanced proliferative activity of PASMCs isolated from the IPAH patients, the increased expression of TRPM7 in the pulmonary arteries of the IPAH patients and the direct relaxing effect of OCS on the human pulmonary artery. (A, B) Summaries of the effects of OCS on the proliferative activity of PASMCs isolated from normal and IPAH patients, as evaluated by an MTT assay (A; *n* = 7–8) and a BrdU incorporation assay (B; *n* = 16). The mean value of BrdU incorporation obtained in the presence of 0.1% OCS was assigned a value of 100%. The data are shown in a boxplot. In A, *P < 0.05 or **P < 0.01 vs control (no treatment with OCS), according to an ANOVA followed by Dunnett’s post hoc test. In B, *P < 0.05 vs vehicle according to a paired Student’s *t* test. (C) Representative chemiluminescence images and a summary of the results of the Western blot analysis (*n* = 5) of the expression of TRPM7 in PASMCs derived from normal and IPAH patients. The data are the mean ± S.E.M. ** *P* < 0.01 vs Normal, according to Student’s *t* test. (D) Representative images (*n* = 5) of the fluorescence staining of *α*-smooth muscle actin (*α*-SMA; green), TRPM7 (red) and DAPI (blue) in lung specimens obtained from IPAH patients. (E) Representative traces and summaries of the relaxant effects of OCS and FTY-720 during the contraction induced by 30 nM U46619 in isolated human pulmonary arteries. The traces obtained under 3 different conditions are overlaid. Averaged %inhibition effects of OCS and FTY-720 are summarized in the panels on the right (*n* = 5–6).

In order to obtain further insight into the role of TRPM7 and the therapeutic effects of OCS in human PAH, several investigations were conducted using human specimens (Fig 7). PASMCs derived from IPAH patients (IPAH-PASMCs) exhibited a greater proliferative activity during the 3-day observation period than those derived from normal subjects (Fig 7A, no OCS treatment). OCS suppressed the enhanced proliferation of IPAH-PASMCs in a concentration dependent manner, whereas it had no significant effect on the proliferation of normal PASMCs at concentrations of up to 0.3% (Fig 7A). The IC_50_ value of OCS for the cell proliferation of IPAH-PASMCs was 0.0138%. The BrdU incorporation assay also showed that 0.1% OCS significantly inhibited the proliferation of IPAH-PASMCs (Fig 7B).

The expression of TRPM7 in IPAH-PASMCs was significantly upregulated in comparison to that observed with normal-PASMCs (Fig 7C). Immunofluorescence staining of the lung tissues obtained from the patients with IPAH demonstrated co-localization of TRPM7 and *α*-SMA in the thickened medial and intimal layers of small PAs (Fig 7D).

In ring preparations of the normal human intrapulmonary artery, OCS and FTY-720 induced relaxation in a concentration dependent manner during the sustained contraction induced by 30 nM U46619, a thromboxane mimetic (Fig 7E). Co-treatment with 100 nM FTY-720 augmented the concentration-dependent relaxant effects of OCS on preconstricted ring preparations of the normal human intrapulmonary artery. (Supplementary Fig 5)

## DISCUSSION

The present study demonstrated, for the first time, that OCS, a traditional Chinese medicine, ameliorated the pathological findings of experimental pulmonary hypertension and significantly improved the survival of MCT-induced PH rats. Importantly, the ameliorating effects of OCS were observed when the OCS treatment was started 11 days after MCT injection (ie, after the pathophysiology of PH developed to a significant level). Mechanistically, the therapeutic effect of OCS is suggested to be attributed to inhibition of TRPM7 activity. The ethanol extract of OCS inhibited the endogenous TRPM7-like currents in HPASMCs and the TRPM7-mediated currents in TRPM7-expressing HEK cells. The OCS extract exerted the inhibitory effects on the TGF-*β*2-induced EndoMT and the phosphorylation of STAT3 and Akt induced by IL-6 and PDGF, respectively. These inhibitory effects were consistent with those observed with either the siRNA-mediated knockdown of TRPM7 or treatment with FTY-720, which is known to inhibit TRPM7 activity.^40^ Moreover, the genetic knockout of TRPM7 ameliorated the development of pulmonary hypertension and vascular remodeling in MCTp-induced PH mice. Our findings therefore strongly suggest a crucial role of TRPM7 in the development of experimental pulmonary hypertension and that OCS exhibited therapeutic effects by inhibiting the TRPM7 activity. The present study provides further evidence that supports the clinical relevance of the role of TRPM7 and the therapeutic effects of OCS in human PAH. Namely, TRPM7 is upregulated in IPAH-PASMCs and associated with medial wall thickening in PA. OCS inhibited the enhanced proliferative activity of IPAH-PASMCs and induced relaxation in the human PA. In addition to the therapeutic potential of OCS all of these observations suggest the more general significance of targeting TRPM7 in the treatment of PAH.

In addition to MCT rats, we investigated TRPM7 expression in the lung and right ventricle of the SuHx-induced PH rats. Endothelial expression of TRPM7 was markedly enhanced in the pulmonary arteries of the SuHx-induced PH rats as compared with the control rats. The level of TRPM7 increased in the a-SMA positive fibroblasts of hypertrophied right ventricles in the SuHx-induced PH rats. These results provide a further support that TRPM7 channel contributes to cardiovascular remodeling that occurs in the progression of pulmonary hypertension.

TRPM7 forms a unique ion channel that contains a kinase domain in its C-terminal region.^42^ The TRPM7 channel is highly permeable to Ca^2+^ and Mg^2+^ and contributes to vascular remodeling by promoting inflammation, fibrosis, cell migration and cell proliferation.^21^ Such effects appear to underlie the significance of TRPM7 in the pathogenesis of PAH. However, it is also reported that the inhibition of the TRPM7 channel activity by heterozygous deletion of the kinase domain (TRPM7^+/Δkinase^) was associated with increased cardiac fibrosis, the increased expression of TGF-*β* and cytokines, the increased phosphorylation of smad3, STAT1 and STAT3, and inflammatory cell infiltration in the heart.^43^ The inhibition of the TRPM7 channel activity by waixenicin A or siRNA-mediated knockdown enhanced proliferation and inhibited apoptosis in PASMCs and exacerbated the hypoxia-induced pulmonary hypertension in rats.^44^ Furthermore, the TRPM7 expression was shown to be downregulated in PASMCs derived from PAH.^44^ These observations of previous reports apparently conflict with the observations of our present study. The exact reason for this discrepancy is unclear. Some important differences should be noted in the experimental conditions, including the TRPM7 inhibitors used (OCS vs waixenicin A), the rat PH model (MCT vs hypoxia), and the mode of genetic invalidation (homozygous global knockout vs heterologous deletion of the kinase domain). Differences in the demographics of the PAH patients and the types of PAH enrolled in the studies could contribute to the disparity in the effects of TRPM7 in the PASMCs derived from PAH patients in the present study. All of these factors may result in the differential contribution of TRPM7 to the pathogenesis in PAH and therefore the therapeutic efficacy of its inhibition may be limited to certain types and stages of PAH.

Treatment with OCS ameliorated the RV remodeling and dysfunction (Fig 1). It is conceivable that a part of these effects is secondary to improvement of PH pathophysiology. However, TRPM7 expression was detected in the fibroblast like cells in the RV (Supplementary Fig 1). TRPM7 has been reported to be the major Ca2+-permeable channel in human atrial fibroblasts. It has been reported to plays an essential role in TGF-*β*1-elicited fibrogenesis underlying atrial fibrillation in humans, and in the cardiac remodeling induced by TGF-*β*1 and angiotensin II.^22^ Therefore, the direct effect of OCS on cardiac fibroblasts might also have contributed to a part of the ameliorating effect of OCS on the RV remodeling and function. However, the precise mechanism still remains to be elucidated.

The active ingredients of OCS that exerted the therapeutic effect in the present study remain unidentified. FTY-720 is synthesized by chemical modification of myriocin, a product of *Isaria sinclairii*, a species of entomopathogenic fungus, which is closely related to OCS. FTY-720 exerts an immunosuppressive effect, agonistic action on sphingosine 1-phosphate receptor 1, an antagonistic effect on cannabinoid receptor 1,^15^ an inhibitory effect on phospholipase A2 and ceramide synthases and TRPM7.^16,17^ The present study revealed that the ethanol extract of OCS possesses a TRPM7-inhibiting activity. Not only OCS, but also FTY-720 are partial inhibitor of TRPM7 and its effects cannot be explained solely by the suppression of TRPM7. The similarity between the ethanol extract of OCS and FTY-720 or TRPM7 knockdown, together with the observations in TRPM7-knockout mice, simply suggest that a part of the therapeutic effects of OCS are mediated by some ingredients in the ethanol extract that inhibit TRPM7 activity. However, because the suspension of the freeze-dried OCS powder was administered to the rats, the contribution of other ingredients of OCS via target molecules other than TRPM7 cannot be entirely ruled out.

How inhibition of TRPM7 exerts the observed effects, such as the inhibition of EndoMT, proliferation, and vasorelaxation, remains unclear. TRPM7 has been reported to contribute to the epithelial-mesenchymal transition in ovarian, prostate, colorectal and breast cancers.^28–33^ Ca^2+^ influx and the subsequent activation of STAT3 or Akt in breast and ovarian cancers, respectively, and Mg^2+^ influx in prostate cancer have been suggested to support the TRPM7-mediated epithelial-mesenchymal transition.^28,29,32^ Moreover, the TRPM7-mediated phosphorylation and inactivation of myosin II and the resultant reduction of cytoskeletal tension may contribute to the epithelial-mesenchymal transition.^33,45^ Mg^2+^ influx supports the proliferation of vascular smooth muscle cells via the activation of mitogen-activated protein kinases and the inhibition of a cell cycle inhibitor p27^Kip1^.^46^ The observed inhibitory effects of OCS on EndoMT, the proliferation of IPAH-PASMC and the phosphorylation of smad2, STAT3 and Akt are consistent with these reported cellular effects of TRPM7.

The observed vasorelaxant effect with the ethanol extract of OCS may also contribute to the therapeutic effect of OCS on MCT-induced PH in rats. However, the underlying mechanism remains unclear. The contribution of TRPM7 to vasoconstriction remains a matter of debate.^21,47^ While the TRPM7-mediated Ca^2+^ influx could contribute to vasoconstriction, the TRPM7-mediated Mg^2+^ influx appears to counteract vasoconstriction. Deficiency of intracellular Mg^2+^, in part due to TRPM7 dysfunction, has been shown to be associated with hypertension.^48^ Furthermore, the activation of Gq protein-coupled receptors has been shown to attenuate TRPM7 activity by activating the phospholipase C and facilitating the hydrolysis of phosphatidylinositol 4,5-bisphosphate.^49^ Vasoconstrictors, such as angiotensin II and endothelin-1, could suppress TRPM7 activity. In view of this uncertainty, it would be premature to ascribe the vasorelaxant effect of the ethanol extract of OCS observed in the present study to TRPM7 inhibition. The finding that 100 nM FTY-720, which itself maximally relaxed preconstricted PA rings, additively augmented the degree of PA relaxation by OCS at its all concentrations tested (Supplementary Fig 5), suggest that FTY-720 may involve a distinct target from that of OCS in producing PA relaxation.

TRPM7 serve as cellular sensors for a wide spectrum of physical and chemical stimuli, such as pressure, stretch, shear stress, osmotic changes and vasoactive substances.^21,50^ How TRPM7 is engaged in the pathological situation of PAH remains unclear. Under the pathological conditions of PAH, the PA pressure increases, the PA is stretched, shear stress increases, and the production of vasoactive substances, such as endothelin and serotonin, increases.^23,51^ All of these factors favor the involvement of TRPM7 in the pathogenesis of PAH. Hemodynamic stresses, such as increased PA pressure, stretching of the PA and increased shear stress, are considered to contribute to the development and maintenance, but not the initiation, of PAH. When administered 11 days after MCT injection, OCS exerted the therapeutic effects and improved the survival of the MCT-induced PH rats. This may support more vital roles of TRPM7 in the progression of PAH, rather than its initiation, and therefore medical intervention inhibiting TRPM7 may serve as a novel strategy for treating PAH in its later stages.

Despite significant advancement of the therapeutic strategies during the last 2 decades, pulmonary hypertension remains an incurable disease. The present study proposes OCS, a traditional Chinese medicine, as a new option for the treatment of PAH. OCS ameliorated the pathological changes in an experimental model of PH. TRPM7 is suggested to be one of the therapeutic targets of OCS. OCS inhibited the enhanced proliferative activity of PASMCs derived from patients with IPAH: the TRPM7 expression was upregulated in PASMCs and was associated with medial hypertrophy in patients with IPAH; and OCS exerted vasorelaxation in the human PA. These observations obtained with clinical specimens suggest the potential of the administration of OCS as a new therapeutic strategy for the treatment of PAH.

## Supplementary Material

Supplementary Material

## AT A GLANCE COMMENTARY

Hiraishi K, et al.

### Background

*Ophiocordyceps sinensis* (OCS), an entomopathogenic fungus, is known to exert antiremodeling effects. The present study explored the therapeutic potential of OCS for pulmonary hypertension (PH) by investigating how it acts on endothelial-mesenchymal transition and enhanced proliferation and contractility of pulmonary artery smooth muscle cells (PASMCs), 3 characteristic changes in PH pathogenesis, with animal PH models, cultured PASMCs and biopsy samples from human PH patients.

### Translational Significance

The results indicate that OCS mitigates PH pathology to improve the survival, substantial part of which is likely mediated by TRPM7 channel inhibition, suggesting the therapeutic significance of OCS for PH progression.

## Clinical specimens for histological evaluations:

The clinical study protocol was approved by the Institutional Review Boards of the Faculty of Medicine, Kyorin University, and the Faculty of Medicine, Fukuoka University(approval number: 16–8-21), and the Faculty of Medicine, Kagawa University (approval number: 2019–204).

## Abbreviations:

*α*-SMA: *α*-smooth muscle actin
EMT: epithelial-mesenchymal transition
EndoMT: endothelial-mesenchymal transition
HR: heart rate
HPAECs: human pulmonary artery endothelial cells
HPASMCs: human pulmonary artery smooth muscle cells
IPAH: idiopathic pulmonary arterial hypertension
IL-6: interleukin-6
MCT: monocrotaline
MCTp: monocrotaline pyrrole
OCS: ophiocordyceps sinensis
PDGF: platelet-derived growth factor
PAs: pulmonary arteries
PAAT: pulmonary artery acceleration time
PAH: pulmonary arterial hypertension
PH: pulmonary hypertension
PAECs: pulmonary artery endothelial cells
PASMCs: pulmonary artery smooth muscle cells
p-STAT3: phosphorylated STAT3
RVID: right ventricular internal diameter
RVWT: right ventricular wall thickness
STAT3: signal transducer and activator of transcription 3
TGF-*β*2: transforming growth factor-*β*2
TNF-*α*: tumor necrosis factor-*α*
TRP channels: transient receptor potential channels
TRPM7: transient receptor potential melastatin subfamily member 7
VE-cadherin: vascular endothelial cadherin
VEGF: vascular endothelial growth factor
vWF: Von Willebrand factor
