# Supplementary material for: Substantial involvement of TRPM7 inhibition in the therapeutic effect of Ophiocordyceps sinensis on pulmonary hypertension
Source: Transl Res. Author manuscript; Available in PMC 2022 Jul 1. (PMC9225677; doi:10.1016/j.trsl.2021.03.004)
Supplement: Supplementary Material [file NIHMS1808508-supplement-Supplementary_Material.pdf]

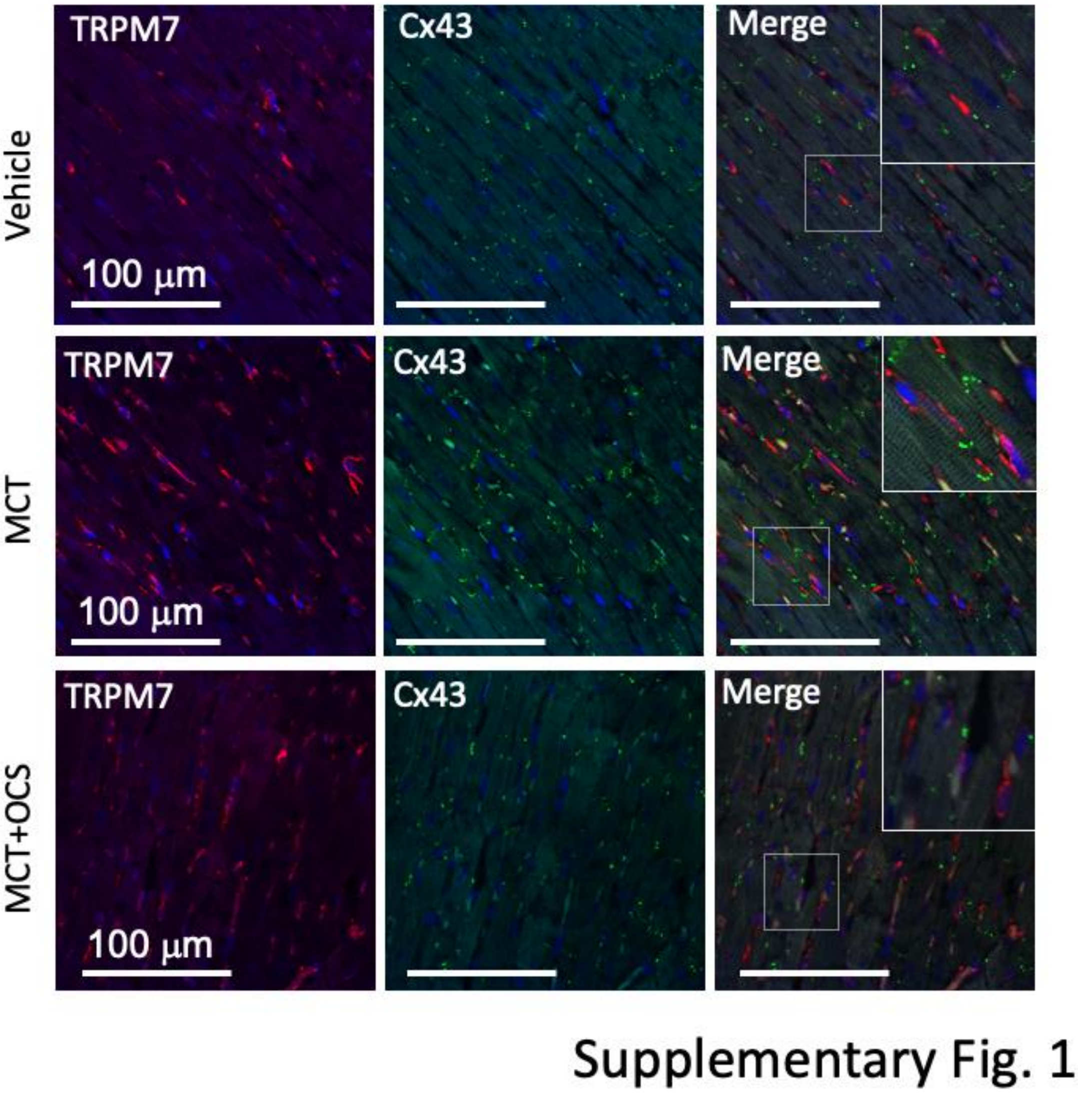

Supplementary Figure 1. Localization of TRPM7 and connexin 43 (Cx43) in the right ventricle (RV) of rats.

Representative images of immunofluorescence staining of TRPM7 (red), Cx43 (green), and DAPI (blue) in the RVs of vehicle, MCT and MCT+OCS rats (n =3).

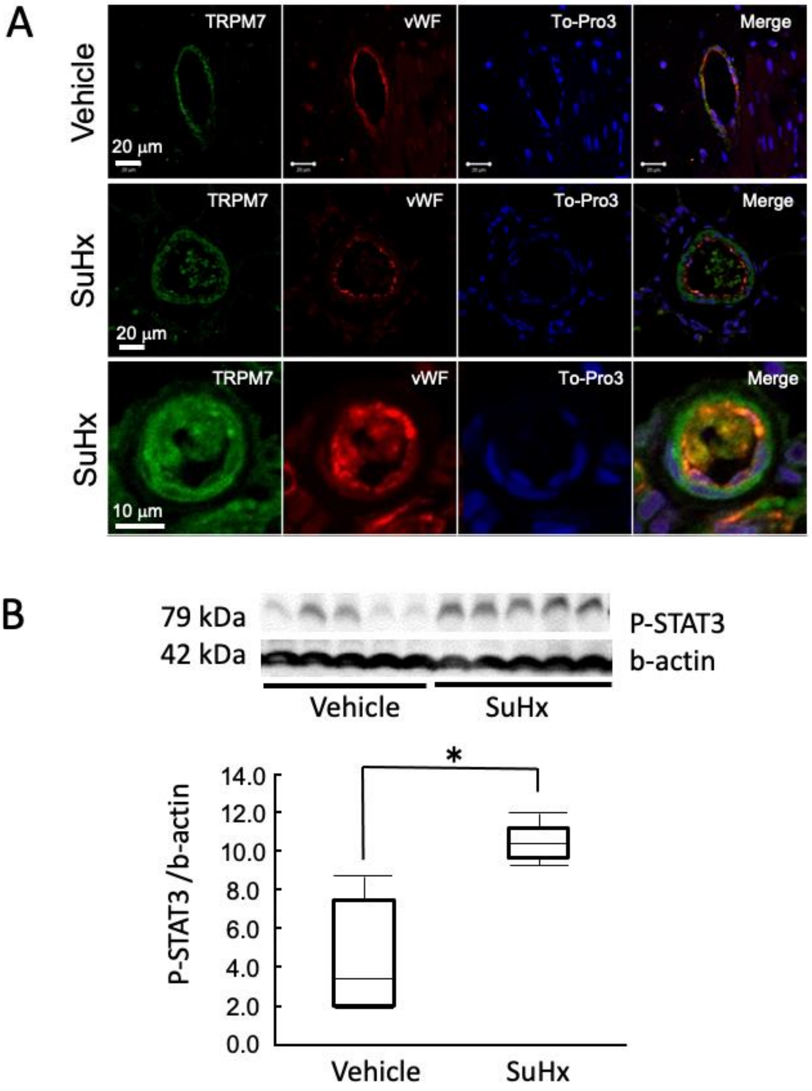

Supplementary Fig. 2

Supplementary Figure 2. Endothelial localization of TRPM7 and increased phosphorylation of STAT3 in the lung of SuHx-induced PH rats. (A) Representative images of immunofluorescence staining of vWF (red), TRPM7 (green) and DAPI (blue) in the lungs of control (CTR) and SuHx-induced PH rats (n =3) (B) Representative image and summary of western blot analysis of p-STAT3 in control and SuHx rats (CTR). ＊ P<0.05 vs. vehicle rats (n =5)

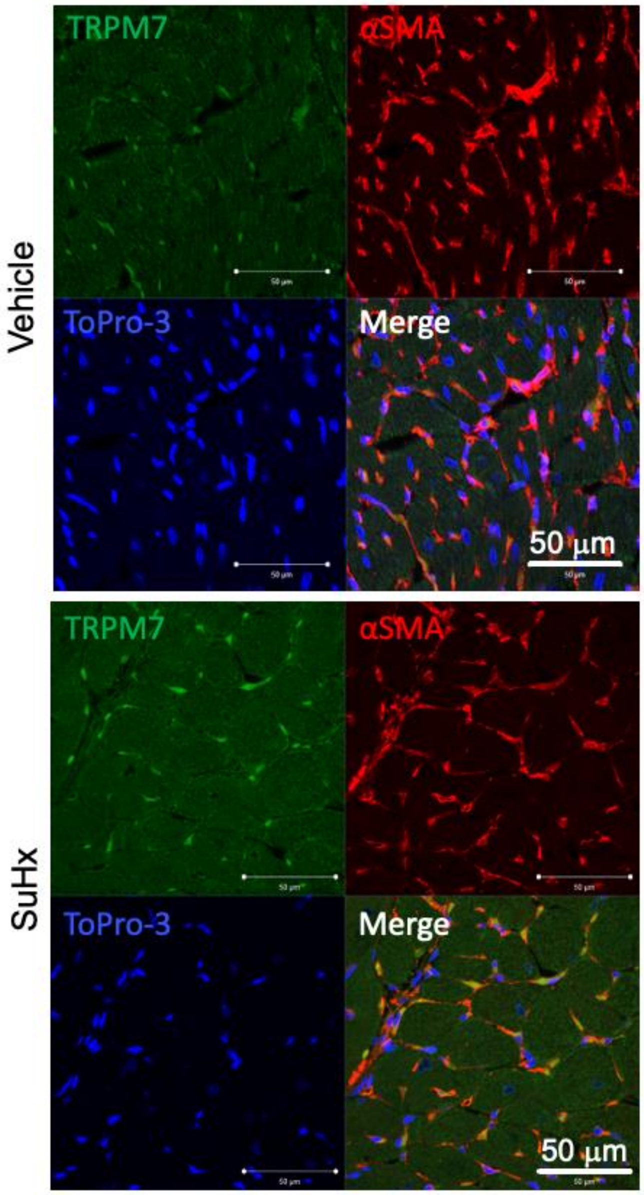

Supplementary Fig. 3

Supplementary Figure 3. Localization of TRPM7 in fibroblasts from a hypertrophied right ventricle of SuHx rat. Representative images of immunofluorescence staining of-SMA (red), TRPM7 (green) and ToPro-3(blue) in the right ventricles of vehicle-treated and SuHx-induced PAH rats (n =4)

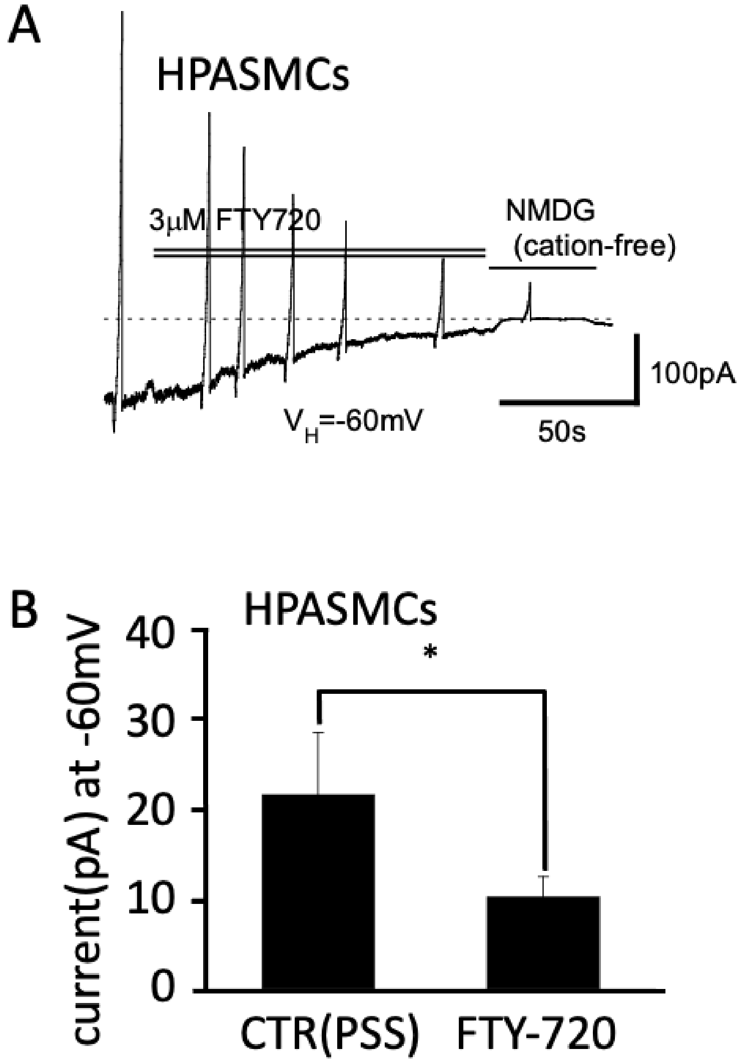

Supplementary Fig. 4

Supplementary Figure 4. Inhibition by FTY-720 of endogenous TRPM7 currents in HPASMCs.

A: Representative recording of an inward current induced by intracellular perfusion of Mg2+-free, ATP-free internal solution in HPASMC, which was significantly inhibited by addition of 3 μM FTY-720. To explicitly define a cation current mediated by TRPM7

channel, NMDG-Cl was perfused at the end of the trace to determine the baseline. Vertical deflections represent currents induced by ramp voltages (from -100 to 100mV, 1s in duration). B: summary for the inhibitory effects of FTY-720 (3 μM). The data are expressed as the mean ± S.E.M. (n = 17). ＊P<0.05 by paired t-test.

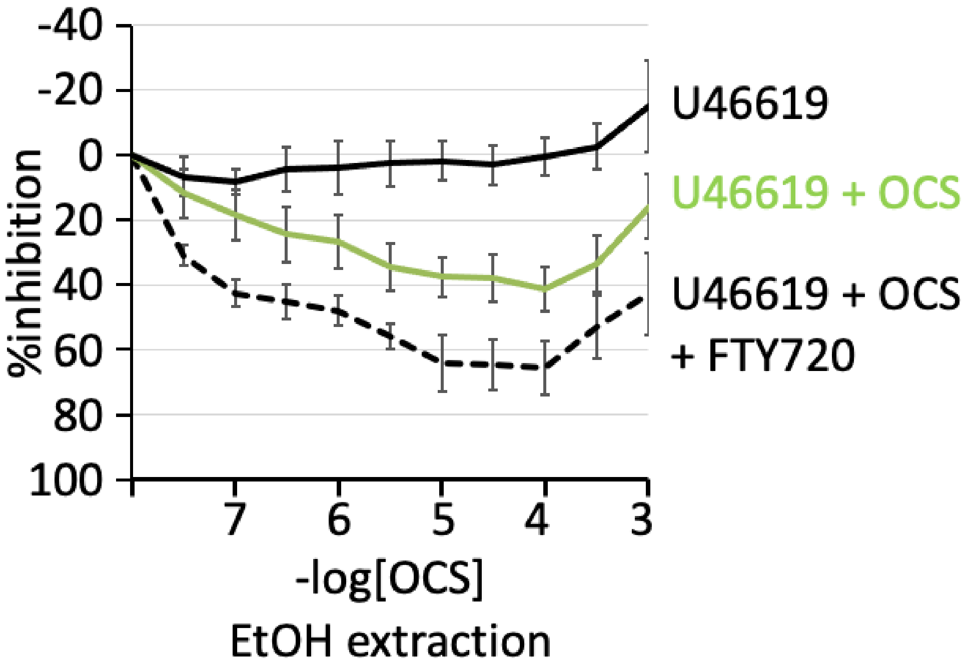

Supplementary Fig. 5

Supplementary Figure 5. Direct relaxing effect of OCS+FTY-720 on the human pulmonary artery

Summaries of the concentration-dependent relaxant effects of OCS in the presence and absence of 100 nM FTY-720 during the contraction induced by 30 nM U46619 in the isolated human pulmonary arteries. Averaged %inhibition effects are summarized as the mean ± S.E.M. (6 experiments from the lungs of 3 patients).

ID	043L	0114F	229J	252V	257X
sampling date	2015/4/15	2014/4/22	2015/2/19	2016/10/19	2018/1/15
age	40	34	53	21	38
sex	male	male	male	male	female
PH	IPAH	IPAH	IPAH	IPAH	IPAH

ID	A15-15	A16-15	A17-7	A18-1	A18-2
sampling date	2015/11/19	2016/9/8	2017/5/2	2018/1/11	2018/1/5
age	59	56	42	89	68
sex	male	male	male	female	female
Disease	Hepatocellular carcinoma	Hepatocellular carcinoma	Superior mesenteric artery thrombosis	Liver cancer	Aortic dissection

Supplementary Table. 1
